# Identification and Validation of a Diagnostic and Prognostic Multi-Gene Biomarker Panel for Pancreatic Ductal Adenocarcinoma

**DOI:** 10.3389/fgene.2018.00108

**Published:** 2018-04-05

**Authors:** Hagen Klett, Hannah Fuellgraf, Ella Levit-Zerdoun, Saskia Hussung, Silke Kowar, Simon Küsters, Peter Bronsert, Martin Werner, Uwe Wittel, Ralph Fritsch, Hauke Busch, Melanie Boerries

**Affiliations:** ^1^Institute of Molecular Medicine and Cell Research, University of Freiburg, Freiburg, Germany; ^2^German Cancer Research Center, Heidelberg, Germany; ^3^German Cancer Consortium, Freiburg, Germany; ^4^Institute for Surgical Pathology, Medical Center – University of Freiburg, Freiburg, Germany; ^5^Comprehensive Cancer Center Freiburg, Freiburg, Germany; ^6^Faculty of Medicine, University of Freiburg, Freiburg, Germany; ^7^Department of Medicine I, Hematology, Oncology and Stem Cell Transplantation, Freiburg, Germany; ^8^Department of Surgery, Faculty of Medicine, Medical Center – University of Freiburg, Freiburg, Germany; ^9^Lübeck Institute of Experimental Dermatology – Institute for Cardiogenetics, Lübeck, Germany

**Keywords:** PDAC, pancreatic precursor lesions, biomarker, meta-analysis, survival, liquid biopsy, ELISA

## Abstract

Late diagnosis and systemic dissemination essentially contribute to the invariably poor prognosis of pancreatic ductal adenocarcinoma (PDAC). Therefore, the development of diagnostic biomarkers for PDAC are urgently needed to improve patient stratification and outcome in the clinic. By studying the transcriptomes of independent PDAC patient cohorts of tumor and non-tumor tissues, we identified 81 robustly regulated genes, through a novel, generally applicable meta-analysis. Using consensus clustering on co-expression values revealed four distinct clusters with genes originating from exocrine/endocrine pancreas, stromal and tumor cells. Three clusters were strongly associated with survival of PDAC patients based on TCGA database underlining the prognostic potential of the identified genes. With the added information of impact of survival and the robustness within the meta-analysis, we extracted a 17-gene subset for further validation. We show that it did not only discriminate PDAC from non-tumor tissue and stroma in fresh-frozen as well as formalin-fixed paraffin embedded samples, but also detected pancreatic precursor lesions and singled out pancreatitis samples. Moreover, the classifier discriminated PDAC from other cancers in the TCGA database. In addition, we experimentally validated the classifier in PDAC patients on transcript level using qPCR and exemplify the usage on protein level for three proteins (AHNAK2, LAMC2, TFF1) using immunohistochemistry and for two secreted proteins (TFF1, SERPINB5) using ELISA-based protein detection in blood-plasma. In conclusion, we present a novel robust diagnostic and prognostic gene signature for PDAC with future potential applicability in the clinic.

## Introduction

Pancreatic ductal adenocarcinoma (PDAC) remains one of the most difficult-to-treat malignancies with a dismal 5-year survival rate of only 5–7% (Siegel et al., 2015). Despite some progress over the last decade, systemic chemotherapy for disseminated PDAC has overall limited efficacy and significant toxicity, while PDACs have been notoriously resistant to molecularly targeted agents and immunotherapy. At the same time, PDAC is projected to become the second leading cause of cancer related deaths in the United States and Europe by 2030 (Rahib et al., 2014), underscoring the pressing need to develop more successful strategies tackling pancreatic cancer (Misek et al., 2007; Siegel et al., 2015).

Given the bleak prospects of clinically manifest PDAC, early detection at the pre-metastatic stage remains a major goal of translational research efforts (Chari et al., 2015). PDACs develop from precursor lesions including pancreatic intraepithelial neoplasia (PanIN) (Guo et al., 2016; Ying et al., 2016), intraductal papillary mucinous neoplasms (IPMNs), and mucinous cystic neoplasms (MCNs) (Hruban et al., 2004). Detecting precursor lesions is still challenging but would improve the chance for curative treatment drastically (Distler et al., 2014) and highlights the need of biomarkers for PDAC. Given the rare detection of non-invasive pancreatic precursor lesions, the amount of such data is very limited and complicates the biomarker discovery. Studying late stage PDAC samples, however, might also give insights at the onset of the disease and therefore make them useful in biomarker research.

To date, carbohydrate antigen 19-9 (CA19-9) is the clinically best established blood-based biomarker for PDAC (Goggins, 2005; Goonetilleke and Siriwardena, 2007). However, the marker’s low sensitivity and specificity disallows its application for early detection. Molecular imaging techniques are being developed for detection of PDAC, but their applicability will probably remain limited to high-risk-situations and radiographic signs are similar to the ones of pancreatitis (Munigala et al., 2014).

In the search for PDAC biomarkers, multiple studies have analyzed the transcriptome and proteome of pancreatic cancer patient tissues (Goonesekere et al., 2014; Bhasin et al., 2016), urine (Radon et al., 2015), and most recently blood samples (Capello et al., 2017). Particularly, the transcriptome displays major differences between PDAC and pancreatic non-tumor tissues, which is an ideal prerequisite to construct a robust PDAC biomarker. PDAC has previously been found to display different tumor subtypes in the transcriptome (Collisson et al., 2011; Moffitt et al., 2015; Bailey et al., 2016). Most recently, Bailey et al. identified four tumor subtypes denominated squamous, ADEX (abnormally differentiated endocrine exocrine), pancreatic progenitor and immunogenic. This underlines the importance of transcriptomics to capture the wide range of responses and corroborates the complexity and variability of the disease as well as the difficulty in developing a comprehensive PDAC biomarker. Transcriptional sensitivity to tumor-normal differences is due to thousands of differentially regulated genes per data set (Badea et al., 2008; Zhang et al., 2012; Haider et al., 2014), however, the small overlap of genes between studies impedes the choice of promising targets and their experimental validation in clinical trials (Harsha et al., 2009). This cohort variability is probably due to varying study designs, different responses of tumor subtypes and/or the transcriptional heterogeneity of PDAC between patients. Therefore, a multi-gene signature resulting from many different studies is required – large enough to capture the multiple facets of the disease and small enough to be applied on individual patient material. Through meta-analysis of multiple data sets a consent of as many as 827 genes have been found to be significantly up-regulated in PDAC (Goonesekere et al., 2014) and as low as five have been previously used for prediction analysis (Bhasin et al., 2016), suggesting meta-analysis as promising tool to identify a gene signature that fulfills the demanded needs.

Here, we present a novel meta-analysis-based approach to identify a robust tissue-based gene signature for classification of PDAC through the incorporation of several independent transcriptome studies. Identified genes were clustered based on gene co-expression and annotated with tissue compartment. A survival analysis of the gene clusters in data from The Cancer Genome Atlas (TCGA) revealed their prognostic behavior for PDAC and allowed the reduction to a clinically feasible signature of 17 genes. These genes were then validated in independent cohorts to assess the applicability of the biomarker not only to pancreatic cancer tissues but also its capability to identify pancreatic precursor lesions and discriminate pancreatitis in fresh-frozen (FF) as well as formalin-fixed paraffin embedded tissues (FFPE). Finally, validation in patient-derived material on transcript and protein level, using qPCR and ELISA-based protein detection in blood plasma indicated the translational potential of the described biomarker panel.

## Results

### Multiple Genes Are Required to Classify PDAC in Independent Data Sets

We collected 18 fresh-frozen PDAC (tumor content 15–80%) and 13 pancreatic non-tumor (normal pancreatic tissue distant to tumor side) tissues and analyzed their transcriptomes using Illumina humanRef-12 bead arrays. After preprocessing and filtering, 21168 genes were further analyzed. Gene set enrichment analysis (Luo et al., 2009) using ConsensusPathDB (Kamburov et al., 2009) identified multiple pathways altered in PDAC samples associated with the development and progression of epithelial malignancies including pancreatic, breast, and lung cancer, confirming the expected pathways of a PDAC cohort and making it suitable for down-stream analysis. Up-regulated pathways included for example the transforming growth factor beta receptor (TGFβR), tumor necrosis factor alpha (TNFα), T-cell antigen receptor (TCR), mitogen-activated protein kinase (MAPK), wingless-related integration site (Wnt) and integrin signaling. In addition, pathways involved in pancreatic secretion were significantly down-regulated (Supplementary Figure S1 and Supplementary Table S1).

**FIGURE 1:**
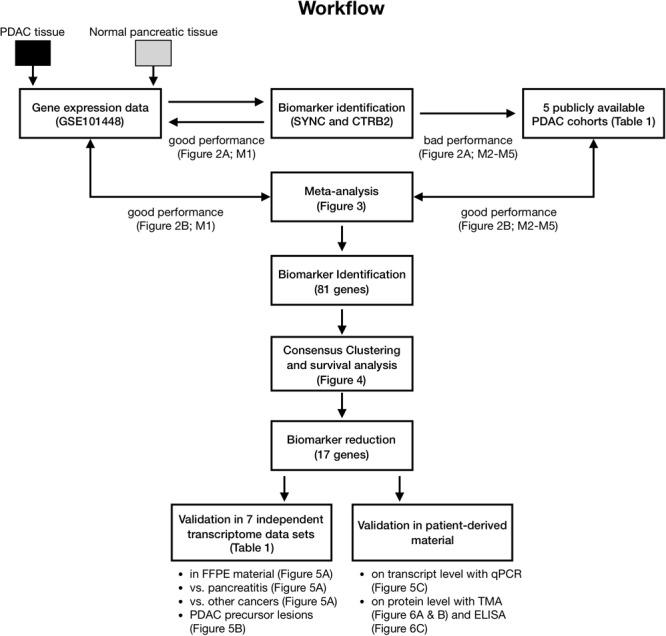
Schematic workflow of analyses.

A schematic flow chart of the performed analysis for biomarker identification is presented in **Figure 1**. First, we sought to identify a minimum predictive gene signature to classify PDAC from non-tumor tissues. Prediction performance was evaluated by 10-fold cross-validation (CV) using a support vector machine (SVM) and features were selected according to log2-fold change (log_2_FC) differences in an inner CV on the training samples to reduce bias through overfitting and to assure independence in the testing set (see Single Set biomarker Identification for M1in Materials and Methods).

**FIGURE 2:**
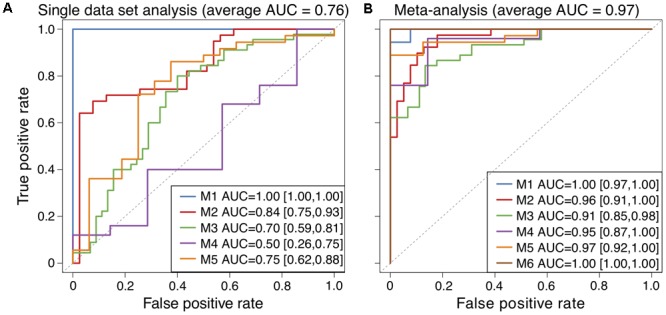
ROC curves to visualize classification performance between PDAC and non-tumor samples. **(A)** Classification performance with two genes (SYNC and CTRB2) selected in inner cross validation of data set M1 (blue line) and validation of these genes in independent data sets M2–M5. **(B)** Classification performances for testing data sets M1–M6 from the meta-analysis. 95% confidence interval of AUC is presented in brackets.

We found two genes, syncoilin (SYNC) and chymotrypsinogen B2 (CTRB2), to discriminate between tumor and non-tumor tissue in data set M1 (**Figure 2A**, blue line). To validate these genes on independent data sets, we selected five publicly available cohorts (Badea et al., 2008; Pei et al., 2009; Donahue et al., 2012; Zhang et al., 2012; Haider et al., 2014) with microarray gene expression data that included a minimum of five samples of both human PDAC and pancreatic non-tumor fresh-frozen tissues (M2–M6, shown in **Table 1**). Cross-validation classification performances with these two genes on M2-M5 (SYNC and CTRB2 were missing in data set M6) revealed poor and highly varying AUCs between 0.50 and 0.84 (**Figure 2A**), clearly indicating overfitting toward our data set, that might arise from sample collection, data preparation, and/or microarray analysis. Therefore, derivation of a small number of classifier genes from a single data set are context-dependent and a more robust gene signature based on multiple data sets is required.

**Table 1:** Summary of data sets used in this study for meta- and validation analysis, including data identifiers, tumor and non-tumor samples and tissue type [fresh-frozen (FF) or formalin fixed paraffin-embedded (FFPE)], feature size after pre-processing, microarray chip, original reference and public repository.

	ID	Pancreatic tumor samples	Pancreatic non-tumor samples	Size	Platform	Reference	Source
Meta-analysis	M1	18 FF PDAC tissues	13 FF tissues	21168	Illumina	–	In house: GSE101448
	M2	36 FF PDAC tissues	36 FF paired tissues	20156	Affymetrix U133plus2.0	Badea et al., 2008	GSE15471
	M3	45 FF PDAC tissues	45 FF paired tissues	18232	Affymetrix HumanGene1.0ST	Zhang et al., 2012	GSE28735
	M4	25 FF PDAC tissues	7 FF tissues	20156	Affymetrix U133plus2.0	Donahue et al., 2012	GSE32676
	M5	36 FF tumor tissues	16 FF tissues	20155	Affymetrix U133plus2.0	Pei et al., 2009	GSE16515
	M6	28 FF PDAC tissues	7 FF tissues	13625	Affymetrix HumanExon1.0STv2	Haider et al., 2014	GSE56560
Validation-analysis PDAC	V1	6 FFPE PDAC tissues	6 FFPE tissues	21168	Illumina	–	In house: GSE101448
	V2	3 FF PDAC and 3 FFPE PDAC tissues	3 FF tissues, 1 FFPE tissue and 10 FFPE pancreatitis tissues	21163	Illumina	–	In house: GSE101462
	V3	Microdissected: 14 FF PDAC	11 FF tissues	17936	Affymetrix U133a and U133b	Grützmann et al., 2004	E-MEXP -950
		Microdissected: 6 FF PDAC and 11 FF adjacent stroma tissues	6 FF tissues and 9 FF pancreatitis tissues	17936	Affymetrix U133a and U133b	Pilarsky et al., 2008	E-MEXP -1121
	V4	178 FF pancreatic cancer tissues	405 bladder, 1080 breast, 280 colon, 369 liver, and 496 thyroid cancer FF tissues	19722	RNA-seq IlluminaHiSeq2000	–	TCGA
Validation-analysis precursor lesions	V5	4 FF PDAC and 13 FF PanIN-2/3 lesions tissues	3 FF pancreatic healthy donor tissues	17936	Affymetrix U133a and U133b	Crnogorac-Jurcevic et al., 2013	GSE43288
	V6	3 IPMNs, 6 IPMAs and 6 IPMCs FF tissues	7 normal pancreatic duct FF tissues	20546	Affymetrix U133plus2.0	Hiraoka et al., 2011	GSE19650
	V7	3 PDAC and 3 PanIN FF tissues from Pdx1-cre; KrasLSL^G12D^ mice	3 FF tissues from Pdx1-cre; KrasLSL^G12D^ mice	20917	Affymetrix mogene10sttran scriptcluster	Ling et al., 2012	GSE33322

**FIGURE 3:**
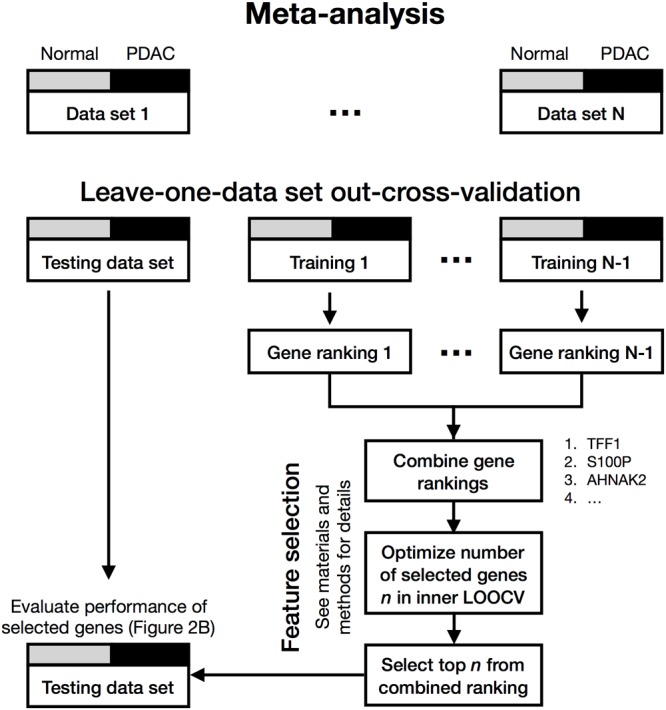
Meta-analysis workflow of N gene expression data sets with two conditions (gray and black). One data set is held out for testing (e.g., M1), while the others (e.g., M2–M6) are used for feature selection. First, for each training data set gene rankings are calculated and subsequently combined. Secondly, the number of selected genes *n ∈* {5,10, …, 50} is optimized by evaluating their overall prediction performance in an inner LOOCV of the training data sets. Then *n* with the best prediction performance is chosen and the top *n* genes are selected from the combined ranking. Finally, the prediction performance for the selected genes of the testing data set is evaluated in a CV with SVM classification (see **Figure 2B**). This is repeated until every data set (M1–M6) was left out for testing once. The selected genes in each round combine to a total of 81 unique classifier genes (Supplementary Table S2). A detailed description of the meta-analysis can be found in the “Meta-Analysis Biomarker Identification for M1–M6” in Materials and Methods.

To derive a cross-study biomarker, we first rendered data sets M1–M6 comparable through application of a meta-analysis algorithm (**Figure 3**). Meta-analysis avoids batch effects arising from different study designs and/or microarray platforms by performing analyses within each data set and not comparing samples across different studies. Briefly, data sets (M1–M6) were split in a leave-one-out-cross-validation (LOOCV) into testing (hold out data set) and training sets, with the latter being used for feature selection. Within the feature selection, the algorithm ranked the genes according to their absolute log_2_FC within each training set, aggregated the rankings and optimized the number of genes *n* that were selected from the combined ranking in an inner LOOCV (see Meta-Analysis Biomarker Identification for M1–M6 in Materials and Methods). The classification results for data sets M1–M6, being held out as testing sets, revealed performances with AUCs between 0.91 and 1.0 and resulted in an average prediction performance of AUC_average_ = 0.97 (**Figure 2B**). The optimal number of classifier genes per data set ranged between 35 and 50, depending on which data sets were used for feature selection. The union of all classifier genes comprised a total of 81 unique genes (Supplementary Table S2). Additionally, we tested other feature rankings, such as *p*-value or SVM weights, but both resulted in larger final gene lists (96 and 134) at equal or worse prediction performance (Supplementary Figures S2A,B). A control run with rearranged class labels led to random prediction performances with an AUC = 0.5 within a 95% confidence interval, which indicated the absence of any bias by the meta-analysis (Supplementary Figure S2C). Taken together, the average prediction performance improved by ΔAUC = 0.21 compared to a single-study analysis (AUC_average_ = 0.76) emphasizing the robustness of the selected classifier genes and the need of considering multiple data sets in biomarker identification.

### Classifier Genes Originate From Distinct Tissue Compartments and Tumor-Specific Subtypes

**FIGURE 4:**
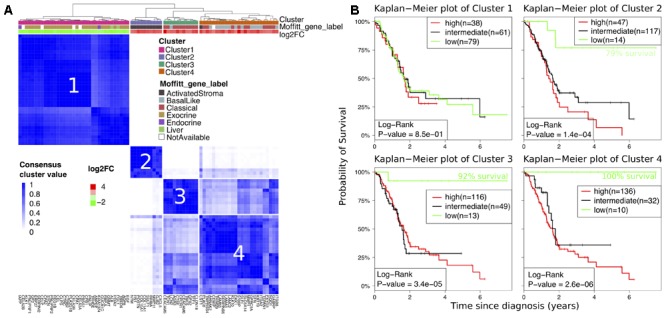
**(A)** Heatmap of consensus matrix from consensus clustering of 81 classifier genes. Cluster membership, gene set labels according to Moffitt et al. (2015) as well as the average log2FC between tumor and normal tissues (M1–M6) are depicted in the annotation bars on the top. **(B)** Kaplan–Meier plots for TCGA data with patient grouping based on low, intermediate and high gene expression of genes in clusters 1–4 (Supplementary Figure S3).

Consensus clustering of the expression values of the 81 classifier genes across the data sets M1–M6 resulted in four distinct clusters (**Figure 4A**). We then functionally annotated the classifier genes according to the gene labels from Moffitt et al. (2015), where available (76 out of 81; Supplementary Table S2). In this study, the authors used non-negative matrix factorization on cancerous and normal pancreatic samples to assign 19749 genes to one of 14 distinct gene labels according to function or tissue origin. Gene labels included normal compartments (distant organs, normal pancreas) and tumor compartments (stroma, tumor subtypes). Interestingly, six gene labels from Moffitt et al. (2015) overlapped significantly with the four clusters identified from consensus clustering (χ^2^ test; *p* < 2e-16; **Figure 4A**).

In detail, 27 of 35 genes in cluster 1 were labeled as exocrine pancreas, including, e.g., pancreatic lipase-related proteins (PNLIPRP) 1 and 2 and serpine family 1 member 2 (SERPINI2). Two genes were labeled as endocrine pancreas: islet amyloid polipeptide (IAPP) and aryl-hydrocarbon receptor repressor (KIAA1234), one gene, carboxypeptidase B1 (CBP1), was assigned to liver, and four genes had no annotation (**Figure 4A**). Furthermore, all genes related to cluster 1 were down-regulated in PDAC compared to non-tumor tissue.

In contrast, all genes in clusters 2–4 were up-regulated in tumor versus non-tumor tissue (log2FC > 0). Genes (*n* = 10) in cluster 2 were entirely labeled as activated stroma (**Figure 4A**), containing periostin (POSTN), fibronectin 1 (FN1) and collagens (COL10A1, COL11A1), which have already been identified as deregulated in precursor lesions of pancreatic cancer (Erkan et al., 2012).

Hierarchical clustering indicated the closest similarity in expression of genes in clusters 3 and 4, probably because cluster members were mainly identified as classical and basal-like tumor genes (**Figure 4A**). 11 out of 12 genes in cluster 3 and 10 out of 26 genes in cluster 4 were labeled as classical tumor genes with the remaining being primarily basal-like genes. The latter include, e.g., laminins and keratins, whereas the former contains adhesion-associated and epithelial genes.

Taken together, consensus clustering and functional annotation of the classifier genes suggest the presence of different biological processes in PDAC progression and their importance for PDAC prediction.

### Classifier Genes Correlate With Patient Survival

Despite an overall poor prognosis, the clinical course of PDAC patients shows remarkable heterogeneity. To examine how gene expression from the four consensus clusters correlates with patient survival, we analyzed survival and gene expression data of 178 pancreatic cancer patients from the cancer genome atlas (TCGA). Hierarchical clustering divided patients into low, intermediate, and high gene expression groups for the individual consensus gene clusters of our biomarker (Supplementary Figure S3).

Indeed, genes in clusters 2–4 were highly associated with survival. Low gene expression groups displayed a 5-year survival rate of 79, 92, and 100%, respectively (**Figure 4B**), while intermediate and high gene expression groups showed significantly worse survival (log-ranks *p* = [0.00014, 0.000034, 0.0000026] in clusters 2–4). Thus, genes in clusters 2–4 have a prognostic significance for pancreatic cancer patients. Contrary to this, there was no survival association among the expression groups in cluster 1 (log-rank *p* = 0.85, **Figure 4B**).

For better clinical applicability, we then sought to reduce the number of genes in the classifier by making use of both the cluster and survival information. Thus, we considered only those genes impacting survival (clusters 2–4) and those that were selected in every hold out data set of the meta-analysis (**Figure 3**), i.e., the most robust genes across the cohorts M1–M6. In total, 17 prognostic classifier genes, selected from the 81 classifier genes, remained for PDAC detection and validation experiments (Supplementary Table S2, marked in yellow).

### Diagnostic Potential of the 17-Gene Classifier in PDAC

Next, we tested the diagnostic potential of the reduced 17-gene classifier for the following four aspects. For clinical application, it is important for (i) the classifier genes to be detectable in formalin-fixed paraffin-embedded (FFPE) samples. Additionally, clinical signs and radiographic findings of PDAC and pancreatitis are often indistinguishable, highlighting the need of a (ii) PDAC biomarker to be insensitive to pancreatitis (Munigala et al., 2014). Another scope of application is (iii) the specificity to PDAC compared to other cancers and last but not least it is beneficial for the classifier genes to be (iv) detectable in pancreatic precursor lesions, which would allow diagnosis at a potentially treatable stage.

To this end, we analyzed the putative clinical prediction performance of the reduced set of classifier genes on seven independent validation transcriptome data sets (V1–V7, **Table 1**). These sets contain gene expression data of fresh-frozen (FF) and FFPE PDAC as well as non-tumor tissues (V1, V2), microdissected PDAC together with its comprised tumor-adjacent stroma (V3), pancreatitis (V2, V3), other cancer entities (V4), and lastly pancreatic precursor lesions of human and mouse (V5–V7).

**FIGURE 5:**
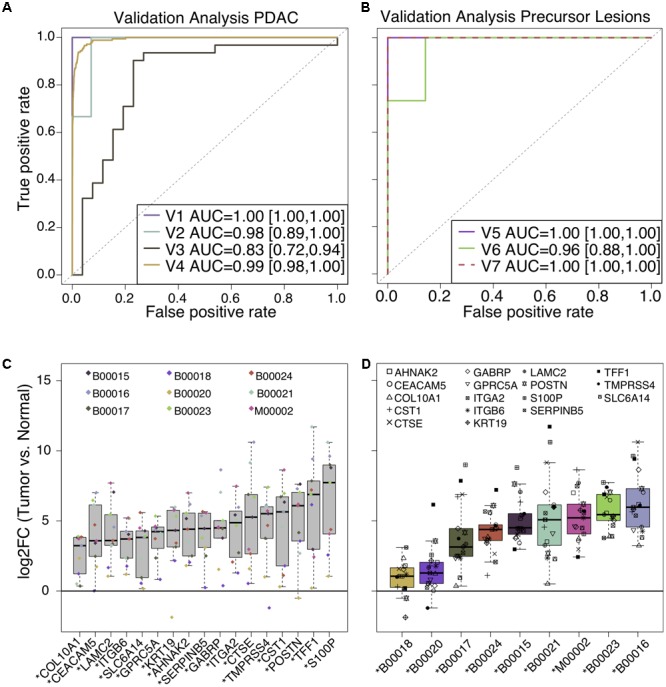
ROC curves of prediction performances of the 17-gene classifier in seven independent data sets (see **Table 1**) for **(A)** validation in PDAC and **(B)** validation in pancreatic precursor lesions. Values in brackets indicate the 95% confidence interval of the AUCs. **(C,D)** qPCR validation of the 17-gene classifier in 8 pancreatic tumors and one liver metastasis. Log2-fold differences between tumor samples and healthy donors are shown sorted by individual genes **(C)** or tumor patients **(D)**.

Prediction of the FFPE sample validation cohort resulted in an optimal separation of PDAC and non-tumor tissues (AUC = 1.00; **Figure 5A**, V1). Data set V2 was comprised of a mix between FF and FFPE samples, including PDAC, non-tumor, and pancreatitis tissues. The 17-gene classifier separated tumor from non-tumor tissues and pancreatitis with an AUC of 0.98, thus confirming its specificity to PDAC and insensitivity to pancreatitis (**Figure 5A**, V2).

Next, we applied the 17-gene classifier on V3, which consists of microdissected epithelial PDAC cells, tumor-adjacent stroma, non-tumor tissue, and pancreatitis, all obtained from FF tissues (Grützmann et al., 2004; Pilarsky et al., 2008). Discriminating tumor (PDAC and adjacent stroma) from non-tumor (normal and pancreatitis) resulted in an AUC of 0.83 (**Figure 5A**, V3).

The tumor-type specificity of the classifier genes was examined by testing the discriminating capability of 178 FF pancreatic cancer tissues compared to >2500 non-pancreatic FF cancer samples (bladder, breast, colon, liver, thyroid) from TCGA. Applying a cross-validation (CV) approach with SVM classification resulted in an AUC of 0.99 (**Figure 5A**, V4), which confirmed the specificity of the classifier genes to pancreatic cancer.

Lastly, we determined the diagnostic potential in early disease stages (**Figure 5B**), whereby we could find only one publicly available study (data set V5). The latter contained FF tissues of three healthy donors, 13 dysplastic PanIN-2 and focal PanIN-3 lesions and four PDAC samples (Crnogorac-Jurcevic et al., 2013). Using CV with SVM classification for our classifier genes, we perfectly discriminated PDAC and PanINs from healthy donors (**Figure 5B**, V5). Because IPMNs are the second major origin of PDAC after PanIN (Hiraoka et al., 2011), we applied the 17-gene classifier to distinguish 15 IPMNs from 7 normal pancreatic duct FF tissues. Again, we used CV with SVM classification and obtained a classification performance of AUC = 0.96 (**Figure 5B**, V6). Because of the vicious cycle of no symptoms at the beginning of the disease and the resulting late diagnosis, early disease stages of human PDAC are very rare. Therefore, we tested the 17-genes in a mouse model to monitor PDAC progression (Ling et al., 2012) for further validation of the detectability of PanIN samples. First, human identifiers were matched to mouse and then normal FF tissues were classified against PanIN and PDAC FF tissues based on the 16 successfully matched genes (excluding S100P), which resulted in optimal separation (AUC = 1.00, **Figure 5B**, V7) using CV analysis.

In conclusion, we could correctly classify PDAC, tumor-adjacent stroma, and pancreatic precursor lesions while classifier genes were not significantly altered in pancreatitis or cancers of distinct origin in FF and FFPE tissues.

### Validation of Classifier Genes on Protein and Transcript Level in Patient-Derived Material

To confirm the gene expression obtained from microarrays, we tested the 17-gene classifier by RT-qPCR technology in biopsies from 8 PDACs and one liver metastasis. mRNA was extracted from fresh-frozen cancerous (*n* = 9) and non-tumor (*n* = 2) pancreas tissues. On average, all biomarker transcripts were significantly up-regulated in tumor compared to non-tumor tissues (one-sided *t*-test: *p*-value < 0.005; **Figure 5C**). When looking at every patient/sample not all of the 17 genes were found up-regulated, however, the set of all classifier genes was always significantly up-regulated in cancerous versus healthy biopsies (one-sided *t*-test: *p*-value < 0.005; **Figure 5D**), corroborating the need for a multi-gene instead of a single gene biomarker for detecting PDAC in individual patients.

**FIGURE 6:**
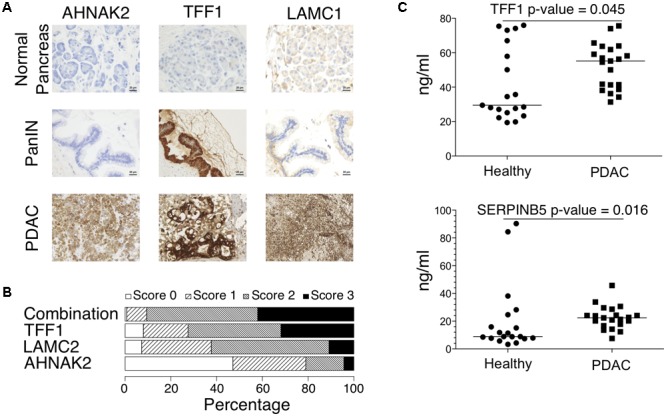
**(A)** Immunohistochemical staining for AHNAK2, TFF1 and LAMC2 in normal pancreas, PanIN and PDAC patients. **(B)** Summary of immunohistochemistry scores for AHNAK2, LAMC2, TFF1 and a combined marker of all of them from 138 patients with pancreatic cancer. **(C)** ELISA-based protein detection in the blood plasma of healthy donors (*n* = 19) and PDAC patients (*n* = 21) for exemplary selected two classifier genes (TFF1, SERPINB5).

Next, we tested the translation of the classifier genes on protein level using tissue microarrays (TMAs) from 138 pancreatic cancers for immunohistochemical staining. **Figure 6A** shows a representative staining for 3 classifier genes: AHNAK2, LAMC2 (cluster 4), and trefoil factor 1 (TFF1, Cluster 3), which were chosen due to their robustness for each cluster. We omitted the analysis of POSTN, the most robust gene in cluster 2, as it has been confirmed recently by immunohistochemical staining to be increased in PDAC compared to non-tumor tissues and correlating with disease progression and poor survival (Liu et al., 2017). We observed a strong positive staining for TFF1 in the PanIN state (score 3) and a strong staining for all markers in the PDAC samples (score 3). An overview of the TMA scores shows that TFF1, LAMC2 and AHNAK2 can be detected in pancreatic cancer samples with a score > 0 in 92, 93, and 53% of all samples, respectively, whereas at least one of the markers was detectable with a score > 0 in 99% of the samples (**Figure 6B**).

Based on the discriminative potential of the above proteins, we investigated the possibility of detecting secreted protein candidates also in plasma samples. We selected exemplary TFF1 and serpin family B member 5 (SERPINB5), which are both secreted, and quantified their abundance using commercially available ELISA assays in plasma samples of PDAC patients. We found significantly elevated levels of these proteins (**Figure 6C**) in PDAC blood plasma samples (*n* = 21) compared to healthy donors (*n* = 19), although some healthy donors displayed elevated levels of both proteins.

Taken into account the to date small sample sizes, we demonstrated the possibility of a promising work-flow (**Figure 1**) for the establishment of a transcriptome and potential proteome biomarker based on tissue and blood plasma samples from PDAC patients. A challenge for further studies would be the applicability of biomarker genes as early detection markers, in particular reaching sufficient number of patients with pancreatic precursor lesions or non-invasive stage PDAC, which again is hindered by the late diagnosis.

## Discussion

The development of robust diagnostic, prognostic and predictive biomarkers for the clinical management of pancreatic adenocarcinoma has been a longstanding objective (Borrebaeck, 2017). We present here a robust gene expression classifier derived through integration of gene expression data from several independent studies. Data integration was achieved via the development of a novel meta-analysis using cross-validation on the level of individual patient cohorts that identified 81 genes for PDAC classification. Our workflow clearly demonstrated the need for a combination of several genes to best stratify PDAC cancer tissue, probably due to the large transcriptional heterogeneity among PDAC patients.

Consensus clustering identified four clusters among the 81 genes that were functionally associated to gene labels assigned by Moffitt et al. (2015) exocrine/endocrine pancreas, classical and basal-like tumor and particularly activated stroma. The latter is in line with the fact that PDAC is heavily interspersed with stromal tissue (Erkan et al., 2012). Additionally, PDAC samples if not microdissected, contain stromal compartments, which are included in the transcriptome analysis and therefore add to the PDAC signature. The PDAC samples used in our data (M1) contained 15–80% of tumor cells, explaining the importance of cluster 2. The exocrine/endocrine pancreas related genes (cluster 1) were all down-regulated in tumor tissues, indicating the functional decline in tumor-associated pancreas tissue. Furthermore, cluster 1 did not correlate with survival and thus turns out to be unusable to monitor the disease. Contrary to this, high and intermediate expression of genes related to stroma (cluster 2), classical (cluster 3) and basal-like (cluster 4) tumor correlated significantly with bad prognosis, making them not only diagnostic, but also prognostic biomarkers. Interestingly, patients with low expression of genes in clusters 2–4 show a highly improved 5-year survival rate (>79%) compared to the expected rate of 5–7% (Siegel et al., 2015), indicating a possibly curable stage of PDAC. This makes these genes not only a diagnostic, but also a prognostic biomarker. However, further research is needed to evaluate, at which expression level the chance of treatment response is promising.

Considering only genes within clusters with a prognostic signature on survival and those being robustly detected within the meta-analysis resulted in a manageable subset of 17 genes. We found multiple genes within the reduced 17-gene classifier that point to a dysregulation of the extracellular matrix (ECM) function in PDAC. The integrin receptors ITGA2 and ITGB6 transduce cell-cell and cell-ECM signaling, whereas POSTN, COL10A1, LAMC2, SERPINB5 and CEACAM5 bind to the integrin receptors and promote cell survival, proliferation, cell adhesion, and migration (Lu et al., 2012), processes which are also important in tumorigenesis.

Further genes are TFF1, known to induce metastasis and to regulate cancer-stroma interactions (Arumugam et al., 2011) and S100P, which is associated with cell proliferation and survival, and is expressed in about 50% of pancreatic lesions (Hu et al., 2014). TMPRSS4 and AHNAK2 promote epithelial to mesenchymal transition (Shankar et al., 2010; de Aberasturi and Calvo, 2015), while GABRP has been linked to PDAC progression and development (Takehara et al., 2007). Lastly, KRT19 and GPRC5A can act as tumor suppressors in other cancers, but have pro-oncogenic functions in PDAC (Ju et al., 2013; Zhou et al., 2016).

With respect to clinical relevance and applicability, we showed that our 17-gene classifier successfully discriminated tumor from non-tumor tissues both in FF and FFPE samples and singled out pancreatitis samples. Most importantly, it correctly classified pancreatic precursor lesions, such as PanINs and IPMNs. Identification and molecular monitoring of preinvasive precursor lesions of PDAC, is of critical importance toward increasing cure rates (Distler et al., 2014). However, material for biomarker development is sparse and often not well categorized mainly because most patients are diagnosed at advanced PDAC stages making extensive validation hard due to a lack of early detection samples. Despite our 17-gene classifier resulting from PDAC patients, it correctly classified pancreatic precursor lesions, such as PanINs and IPMNs, encouraging the transition to early stages.

Recently, Bhasin et al. (2016) published a 5-gene classifier where 4 genes overlapped with our reported 17 genes (AHNAK2, SERPINB5, TMPRSS4 and POSTN), and epithelial cell transforming 2 (ECT2) was unique to their analysis. They selected genes that were differentially regulated in three out of four data sets and tested gene combinations ranging from 2 to 40 genes for best prediction performance in four training sets, which resulted in the 5 reported genes. In comparison, we arrived at a larger gene list (81) after unifying all suggested classifier genes from a LOOCV of six data sets. Two overlapping data sets, M3 (GSE28735) and M4 (GSE32676), in both studies allowed direct comparison of prediction performances and showed that genes obtained within our meta-analysis scored consistently better than Bhasin et al.’s (2016) (AUCs of 0.95 and 0.91 vs. 0.90 and 0.88 for M3 and M4, cf. **Figure 2B**). Of note, we selected 45 and 40 genes within the feature selection of the meta-analysis in contrast to Bhasin’s five genes, suggesting better performances for more genes.

Additionally, Bhasin et al. (2016) used hierarchical clustering to test their 5-gene classifier on human IPMN (V6) and mouse PanIN samples (V7, **Figure 4B**). While their 5-gene signature separated all but one normal pancreatic duct samples, our 17-gene classifier achieved perfect separation in data set V6 (Supplementary Figure S4A). Both their 5-gene and our 17-gene classifier perfectly discriminated between normal, PanIN and PDAC cases in a genetically engineered mouse model to study PDAC progression (Ling et al., 2012) (Supplementary Figure S4B). However, prediction performance of Bhasin et al.’s (2016) five genes in human PanIN samples (V5) resulted in a significantly worse performance (AUC = 0.82, Specificity = 0) compared to our 17-gene biomarker (AUC = Sensitivity = Specificity = 1). Particularly, the genes TFF1 and S100P separated IPMNs and PanINs from healthy donors, emphasizing their crucial role in detecting pancreatic precursor lesions. While the 5-gene classifier is superior to a single gene biomarker (Bhasin et al., 2016), the results presented here suggest the need to include further genes to increase robustly PDAC stratification. Interestingly, the 5-gene classifier contained only genes labeled related to activated stroma (POSTN) and basal-like tumor (AHNAK2, SERPINB5, TMPRSS4), and excluded classical tumor-like genes, which might explain the inferior classification performance.

In this context, we hypothesize that a widely applicable biomarker should cover the different tumor subtypes as described by the transcriptomic PDAC landscape (Collisson et al., 2011; Moffitt et al., 2015; Bailey et al., 2016). Using Moffitt et al.’s (2015) class labels, the 17-classifier included representatives of the basal-like and classical like tumor subtype as well as the activated stroma. In comparison to Collisson et al.’s (2011) subtypes, we find eleven genes within the 17-gene classifier (AHNAK2, CEACAM5, CTSE, GABRP, GPRC5A, ITGA2, ITGB6, LAMC2, S100P, SLC6A14, TFF1) associated to the classical and one (POSTN) to the quasimesenchymal subtype according to their NMF scores. The exocrine-like subtype corresponded to our cluster 1 (exocrine/endocrine), including eleven representatives such as, PNLIP, PNLIPRP2, and CEL. Bailey et al. identified four different subtypes, squamous, ADEX, pancreatic progenitor, and immunogenic. There were no NMF scores of genes from clustering available, but AHNAK2, POSTN, and LAMC2 were significantly up-regulated (adj.*p*.val < 0.05) in the squamous subtype, and TFF1 and CTSE in the progenitor subtype with respect to the other subtypes, making these genes good subtype representatives. The ADEX subtype was exclusively associated to cluster 1 (exocrine/endocrine) with 26 genes being significantly up-regulated. Interestingly, we did not find any genes significantly up-regulated in the immunogenic subtype. This can be explained by the fact that the immunogenic subtype is mostly represented by immunoglobulin genes, however, they were not included in the probe sets of the investigated microarray chips. Nevertheless, the immunogenic subtype has characteristics similar to the progenitor subtype and is part of Collisson’s classical subtype, which were covered by the 17-gene classifier. Therefore, we capture the entire pancreatic cancer spectrum, with the exception of the exocrine-like (Collisson et al., 2011), the ADEX (Bailey et al., 2016) and the exocrine/endocrine subtype (Moffitt et al., 2015). Representatives of these subtypes were identified in our meta-analysis approach (cluster 1) but neglected in the 17-gene classifier because the genes showed no survival association and were down-regulated in tumor compared to normal pancreas tissue.

Despite the limited number of patients, the classifier yielded significant up-regulation in gene expression of PDAC patients versus healthy individuals, using microarray and RT-qPCR technologies and was clearly present on the protein level by TMA. Using a “liquid biopsy” approach on two exemplary chosen proteins (TFF1, SERPINB5), we found significantly elevated levels in PDAC patient blood samples compared to healthy donors, which might open up new avenues for clinical applicability and robust, minimal-invasive detection of PDAC. For this, however, larger cohort sizes, specificity compared to other cancer types and testing of further classifier-derived proteins will be needed for successful clinical translation.

Taken together, we established a novel meta-analysis pipeline for robust biomarker identification for PDAC versus non-tumor tissues, which can also be applied to different two-group experiments. Subsequent analysis revealed the diagnostic and prognostic influence of the 17-gene signature in PDAC, including pancreatic precursor lesions with application in patient tissues and liquid biopsies presenting a work-flow with potential impact in clinical transition.

## Materials and Methods

### RNA Isolation and Quantitative Real Time PCR (qRT-PCR)

Total RNA was isolated from FF and FFPE tissue samples according to the manufacturer’s protocol and as described by Offermann et al. (2016). For FF tissues the Universal RNA Purification Kit from Roboklon (Germany) and for FFPE tissues the RNeasy FFPE Kit from Qiagen were used. qRT-PCR was performed as described elsewhere (Offermann et al., 2016) and relative gene expression levels were calculated with the 2^-ΔΔC_T_^ method, using HPRT1 and 18S ribosomal RNA as reference genes (primers in Supplementary Table S3). Log2-fold changes were calculated compared to healthy tissue and tested for significant elevation by one-sided *t*-tests compared to log2-fold change = 0.

### Microarray Preprocessing

Total RNA was isolated, labeled and hybridized using the Whole-Genome protocol from DASL HT Assay (Illumina, San Diego, CA, United States) and Ovation^®^ FFPE WTA-Systems (NuGEN, San Carlos, CA, United States) to an Illumina humanRef-12 beadarray (Illumina, San Diego, CA, United States) according to the manufacturer’s protocol. Raw bead count data was analyzed using the R/Bioconductor package beadarray (Dunning et al., 2007) followed by quantile normalization and log2 transformation. Data is accessible on Gene Expression Omnibus (GEO) as GSE101448 and GSE101462. Public microarray data from GEO were downloaded in pre-processed form, i.e., normalized, log2 transformed and filtered on probe level, according to the original reference (see **Table 1**). Since E-MEXP-1121 is a follow-up study of E-MEXP-950, we combined the two data sets from ArrayExpress. HGU133a and HGU133b chips from both studies were normalized by robust multiarray averaging (RMA) and subsequently combined, neglecting the lower inter-quartile range (IQR) if probes were present on both chips. For all data sets platform probe IDs were matched to unique EntrezIDs. In the case of multiple probes matched the same EntrezID, we chose the probe with the largest IQR.

### Gene Set Enrichment Analysis

Enrichment of signaling pathways were performed as implemented in the R/Bioconductor package GAGE (Luo et al., 2009) with ConsensusPathDB pathways (Kamburov et al., 2009). Pathways were considered significant with an adjusted *p*-value < 0.01 (Benjamini-Hochberg) and were connected by an edge if they share 30% of their genes (Supplementary Figure S1).

### Single Set Biomarker Identification for M1

The data set (M1) consisting of tumor and non-tumor samples was iteratively split into testing and training samples in a 10-fold cross-validation (CV). For each testing set, we selected the features in an inner CV of the training samples to reduce the bias of overfitting. First, we obtained absolute log_2_FC differences between tumor and non-tumor tissues (Ritchie et al., 2015) and ranked them accordingly. Secondly, we optimized the number of genes that were selected. This is done by calculating the prediction performance when *n ∈*{1,2, …, 10} genes were selected by applying an inner CV on the training samples. Thirdly, the top *n* genes were selected from the gene ranking, where *n* corresponds to the best prediction performance of the inner CV. These genes were then selected to train a support vector machine (SVM) on the training samples. Finally, the SVM model was used to predict tumor and non-tumor samples in the independent testing set. For all testing sets no more than two genes (SYNC and CTRB2) were sufficient for perfect prediction.

### Meta-Analysis Biomarker Identification for M1–M6

To construct a robust gene signature discriminating between tumor and non-tumor tissues across data sets we applied a leave-one-out-cross-validation (LOOCV) approach on the independent PDAC cohorts (**Figure 3**). For every cross-validation run, one data set was held out for testing while the others (N-1) were used for feature selection.

#### Combine Gene Rankings

First, genes were ranked within each cohort (N-1) according to their absolute differential log_2_FC between tumor and non-tumor tissue (Ritchie et al., 2015). Let *R_ig_* be the rank of gene g in data set i. To adjust for different total number of features, we normalized gene ranks according to the maximum rank of the data set $normR_{ig}=\frac{R_{ig}-1}{\max(R_{i})-1}$. The normalized ranks were then combined by calculating their mean across data sets $aveR_{g}=\frac{1}{N-1}\sum_{i=1}^{N-1}normR_{ig}$ to obtain an overall gene ranking.

#### Optimize the Number of Genes to Be Selected

Secondly, to reduce the bias of overfitting, we used an inner LOOCV on training data sets to optimize the number of genes that were selected from the combined gene ranking. Therefore, we chose *n ∈*{5,10, …, 50} with the best prediction performance in an inner LOOCV across all training data sets (N-1). Then the top *n* genes were selected from the combined gene ranking and used to evaluate the prediction performance of the selected genes.

#### Evaluate Performance of Selected Genes

For a given set of genes we evaluated the prediction performance for the testing data set (in both outer and inner LOOCV) by using a SVM with a Gaussian kernel in a balanced 10-fold CV on tissue samples. SVM parameters (C, γ) were optimized using a grid search in an inner CV (Meyer et al., 2015). The area under the curve (AUC) of the receiver-operating characteristic (ROC) served as performance evaluation metric (Sing et al., 2005).

For every testing data set, between 35 and 50 genes were chosen in the feature selection, which combined to a total of 81 unique classifier genes.

### Consensus Clustering

We used the ConsensusClusterPlus package (Wilkerson and Hayes, 2010) with Ward’s clustering and Spearman correlation distance metric to cluster genes in M1–M6. The optimal number of clusters *k = 4* was obtained by investigating the empirical cumulative distribution function (CDF) and its relative change as proposed by Wilkerson and Hayes (2010) (Supplementary Figures S5A,B).

### Survival Analysis

RMSE normalized gene expression data of 178 PDAC samples and their associated survival information were downloaded from TCGA. Based on the consensus clusters, we applied Ward’s hierarchical clustering on expression data using Euclidean distances to divide patients into low, intermediate and high expression groups (Supplementary Figure S3). Survival rates were analyzed using Kaplan–Meier plots and log-rank tests.

### Validation Analysis

Prediction performances were estimated by 10-fold CV with SVM on validation data sets with classifier genes as features as described “Evaluate Performance of Selected Genes” in “Materials and Methods.” To compare PDAC to other cancers, we obtained RMSE normalized gene expression data from TCGA for bladder, breast, colon, liver, and thyroid cancer.

An overview about the statistical methods used is given in Supplementary Table S4 and the meta-analysis algorithm has been uploaded to: https://github.com/hklett/meta-analysis.git.

### Immunohistochemistry

All patients were treated at the Clinic for General and Visceral Surgery and histopathological work-up was performed at the Institute of Surgical Pathology, both University Medical Center Freiburg, Germany. All tumor samples were reviewed by experienced pathologists. In total, 138 patients were included in tissue microarray (TMA) analysis. Before core biopsy was withdrawn from the donator and inserted into the acceptor paraffin block, tumor tissue was outlined at the corresponding hematoxylin-eosin slide. Each TMA comprised up to 24 patients. All patients were represented by two core biopsies with a core diameter of 2 mm. Hereby, each core biopsy was allocated at a separate TMA-block. Serial 2 μm thick tissue slices were prepared for Estrogen Inducible Protein ps2 (TFF1; ab92377, Abcam plc, Cambridge, United Kingdom), anti-LAMC2 (HPA024638, Sigma-Aldrich, Munich Germany) and anti-AHNAK2 (HPA002940, Sigma Aldrich, Munich, Germany). Slides were dried at 56°C overnight to improve adherence to the objective plate and then deparaffinized in xylene and decreasing ethanol concentrations. For TFF1 and LAMC2, heat mediated epitope retrieval was performed for 20 min at pH 6.1 and 95°C. For AHNAK2 epitope retrieval was not necessary. For all antibodies immunohistochemistry (IHC) was performed using the Autostainer plus and stained afterward with Dako Real^®^ Detection System (Dako K5001) according to the manufacturer’s guidelines. Negative control was performed via omission of primary antibody. Two pathologists blinded for patient data, reviewed Anti-TFF1, Anti-LAMC2 and Anti-AHNAK2 according to the following protocol. Using 200-fold magnification, antibody expression was analyzed in each core biopsy using a semi-quantitative expression analyses for antibody intensity (score 0 = none, 1 = low, 2 = intermediate, 3 = strong) and percentage of positive (range 0–100%, intervals of 5%) tumor cells.

### Enzyme-Linked Immunosorbent Assays

Enzyme-linked immunosorbent assays (ELISA) of TFF1 and SERPINB5 were performed following the manufacturer’s instructions (SEB049Hu, Cloud-Clone Corp. Houston, TX, United States; LS-F13455, LifeSpan BioSciences Inc., Seattle, WA, United States). The antibodies for detection of TFF1 and SERPINB5 were provided with the respective kits. The plasma samples were diluted by a factor of four. Standard or blood plasma samples (100 μl) were pipetted on a provided plate in duplicates and incubated at 37°C for 1 h. TFF1 and SERPINB5 proteins were detected using the provided detection reagents and the plate was read at 450 nm using a TECAN Infinite M200PRO plate reader.

### Ethical Approval and Patient Approval

All study participants have given their written consent and the study was approved by institutional ethics regulations (#126/17; Ethics Commission, Albert Ludwigs University of Freiburg, Germany).

## Author Contributions

HK, HB, and MB conceived the study. SKü and UW performed the surgery and collected the tissue samples for micorarrays. HK, HB, and MB performed the statistical analysis, including the meta-analysis. HF, PB, and MW were involved in tissue storage and TMA analysis. EL-Z, SKo, and MB performed the ELISA analysis. SH and RF provided the patient-derived samples. HK, HB, MB, and RF wrote the manuscript and generated figures and tables. All authors critically revised the manuscript.

## Conflict of Interest Statement

The authors declare that the research was conducted in the absence of any commercial or financial relationships that could be construed as a potential conflict of interest.
