# Supplementary material for: Identification and Validation of a Diagnostic and Prognostic Multi-Gene Biomarker Panel for Pancreatic Ductal Adenocarcinoma
Source: Front Genet. 2018 Apr 5;9:108. doi: 10.3389/fgene.2018.00108 (PMC5895731; doi:10.3389/fgene.2018.00108)
Supplement: Supplementary file 4 [file Table_4.docx]

**Supplementary Table 4** Overview about the algorithms and their underlying statistical methods.

| **Algorithms** | **Statistical methods** |
| --- | --- |
| Gene set enrichment | GAGE (Luo et al. 2009) with pathways from ConsensusPathDB (Kamburov et al. 2009) (Supplementary Figure 1) |
| Meta-analysis | Support vector machine, cross-validation, limma for gene ranking, area under the curve (AUC) of the receiver operating characteristic (ROC) for performance evaluation (Figure 2B and Figure 3) |
| Consensus clustering | Distance metric: 1-Spearman correlation coefficient; Clustering method: Ward’s hierarchical clustering (Figure 4A). 𝛘^2^ – test for cluster association. |
| Survival analysis | Kaplan-Meier plot, log-rank test (Figure 4B) |
| Validation analysis | Support vector machine, cross-validation, AUC for performance evaluation (Figure 5A and B) |
| qPCR and ELISA | qPCR: one-sided t-test (Figure 5C and D); ELISA: two-sided t-test (Figure 6C) |
