# Supplementary material for: Identification and Validation of a Diagnostic and Prognostic Multi-Gene Biomarker Panel for Pancreatic Ductal Adenocarcinoma
Source: Front Genet. 2018 Apr 5;9:108. doi: 10.3389/fgene.2018.00108 (PMC5895731; doi:10.3389/fgene.2018.00108)
Supplement: Supplementary file 5 [file Image_1.PDF]

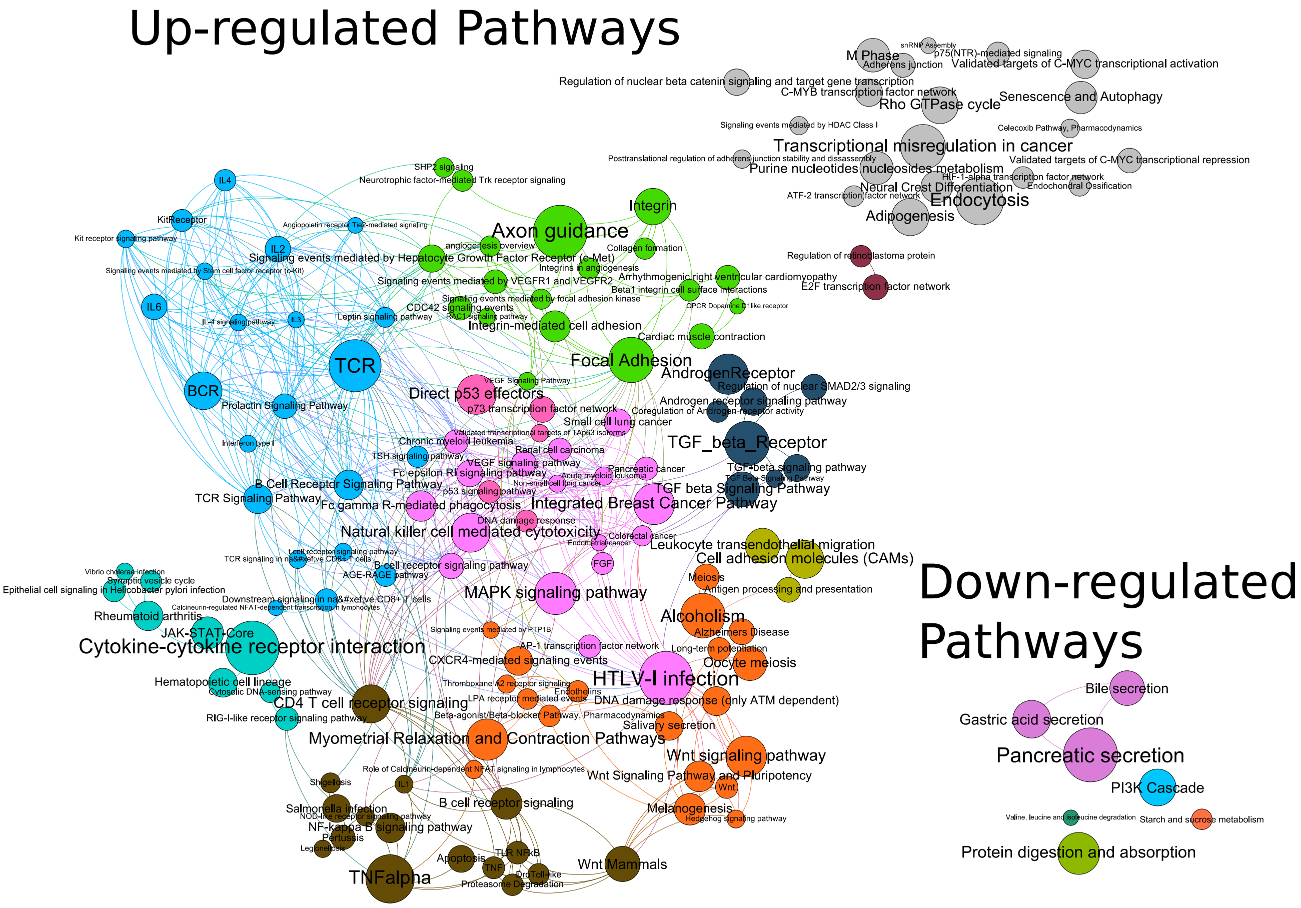

Supplementary Figure 1 Gene set enrichment analysis of PDAC vs. non-tumor samples with ConsensusPathDB. Node diameters correspond to gene set size and different colors are used to highlight communities within the graph.
