# Supplementary material for: Identification and Validation of a Diagnostic and Prognostic Multi-Gene Biomarker Panel for Pancreatic Ductal Adenocarcinoma
Source: Front Genet. 2018 Apr 5;9:108. doi: 10.3389/fgene.2018.00108 (PMC5895731; doi:10.3389/fgene.2018.00108)
Supplement: Supplementary file 6 [file Image_2.PDF]

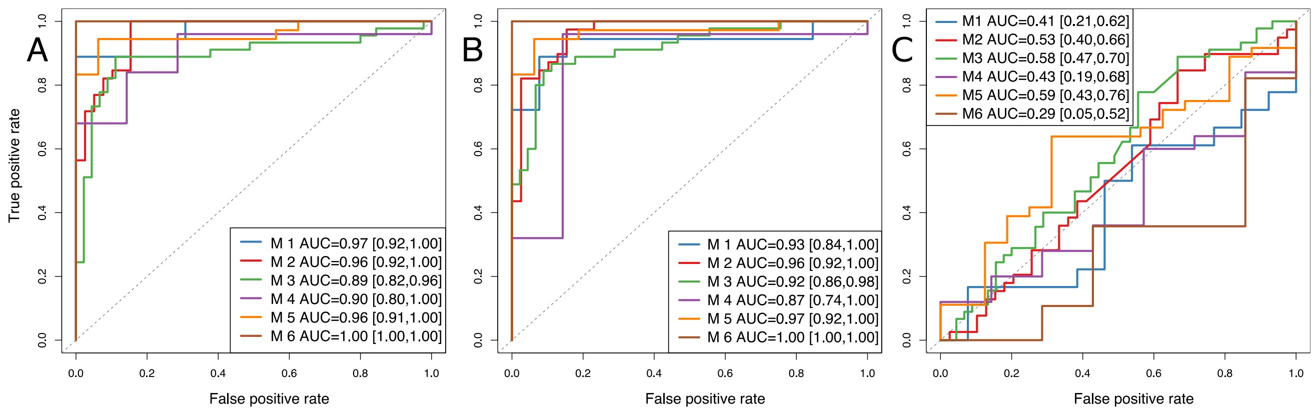

Supplementary Figure 2 ROC curves visualizing the classification performance between PDAC and non-tumor samples. Meta-analysis classification performances from features selected based on p-value (A), SVM weights (B) and randomized class labels (C). Values in brackets indicate the 95% confidence interval of the AUCs.
