# Supplementary material for: Identification and Validation of a Diagnostic and Prognostic Multi-Gene Biomarker Panel for Pancreatic Ductal Adenocarcinoma
Source: Front Genet. 2018 Apr 5;9:108. doi: 10.3389/fgene.2018.00108 (PMC5895731; doi:10.3389/fgene.2018.00108)
Supplement: Supplementary file 7 [file Image_3.PDF]

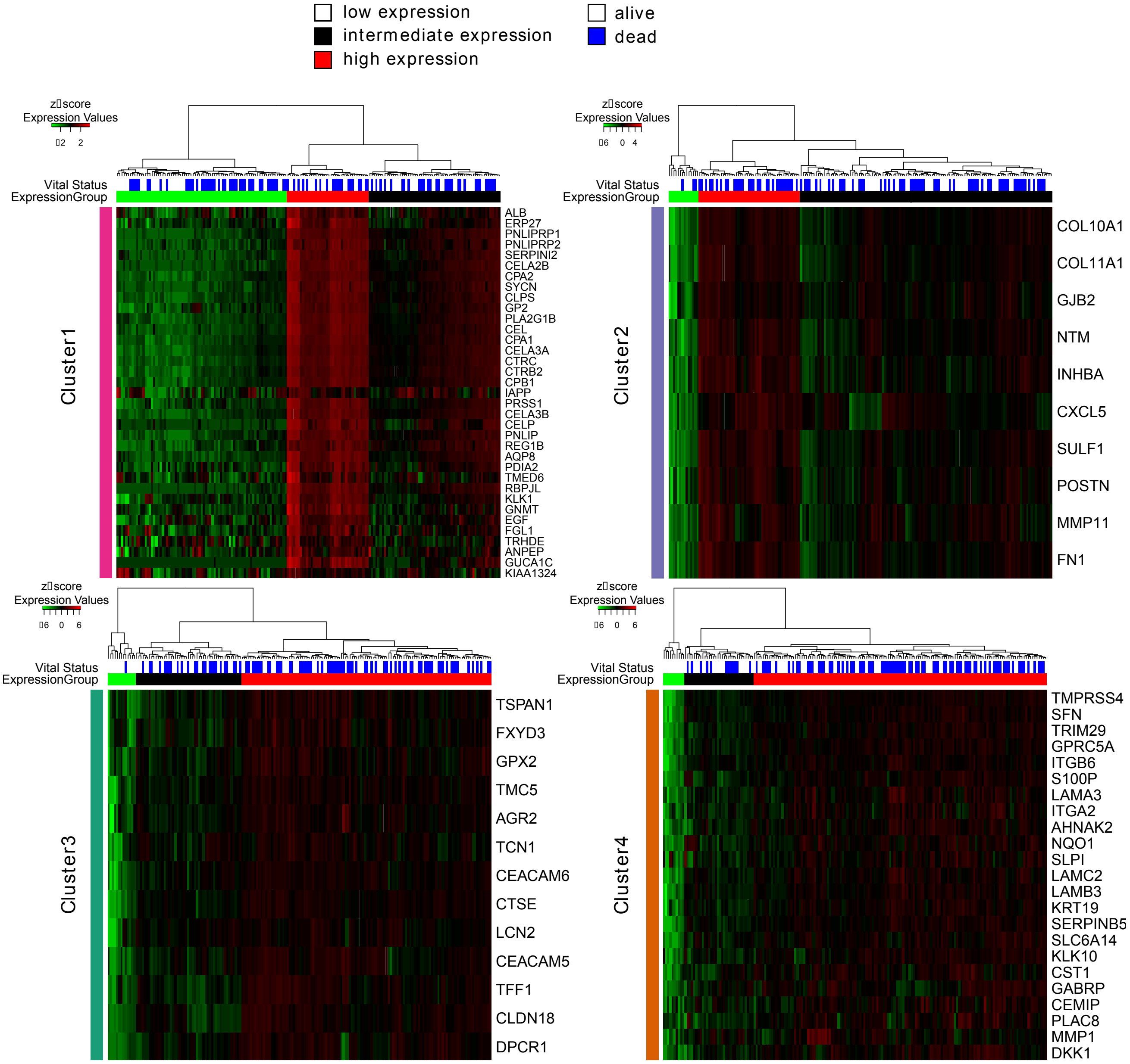

Supplementary Figure 3 Heatmaps with Ward's hierarchical clustering to estimate low (green), intermediate (black) and high (red) expression groups. Vital status is shown in the annotation bar on top with dead or alive indicated as blue or white, respectively. Data taken from TCGA.
