# Supplementary material for: Identification and Validation of a Diagnostic and Prognostic Multi-Gene Biomarker Panel for Pancreatic Ductal Adenocarcinoma
Source: Front Genet. 2018 Apr 5;9:108. doi: 10.3389/fgene.2018.00108 (PMC5895731; doi:10.3389/fgene.2018.00108)
Supplement: Supplementary file 8 [file Image_4.PDF]

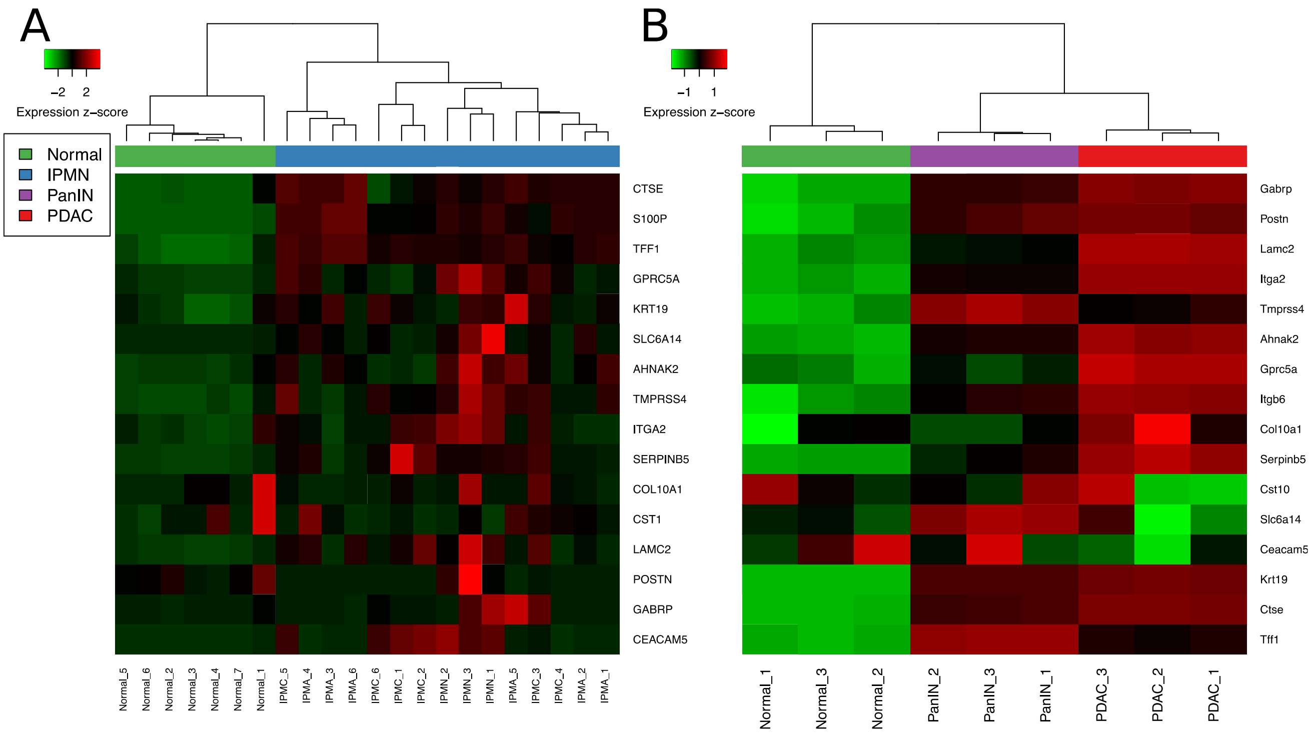

<u>Supplementary Figure 4</u> Heatmaps of gene expression data with complete clustering based on Euclidean distances of the 17-gene classifier. A: Precursors data set (V6; GSE19650) with normal pancreatic duct (green) and IPMN (blue) tissues. B: PDX1-Cre; LSL-Kras^{G12D} data set (V7; GSE33322) with normal (green), PanIN (purple) and PDAC (red) tissue.
