# Supplementary material for: Identification and Validation of a Diagnostic and Prognostic Multi-Gene Biomarker Panel for Pancreatic Ductal Adenocarcinoma
Source: Front Genet. 2018 Apr 5;9:108. doi: 10.3389/fgene.2018.00108 (PMC5895731; doi:10.3389/fgene.2018.00108)
Supplement: Supplementary file 9 [file Image_5.PDF]

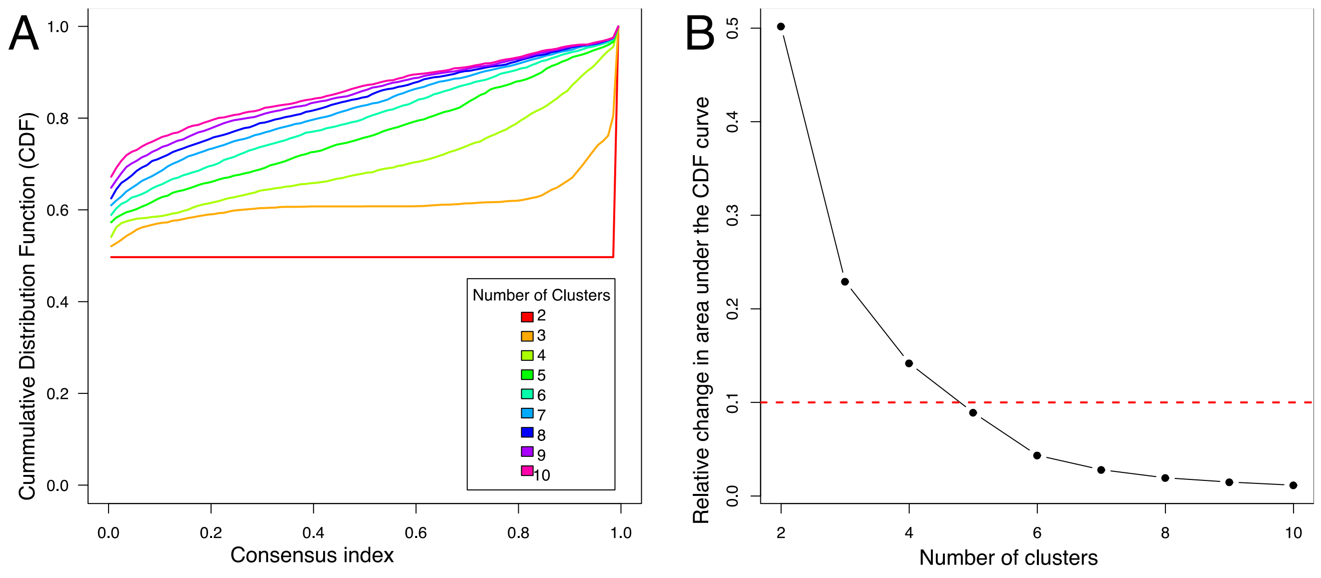

Supplementary Figure 5 A: Cumulative distribution function (CDF) of consensus matrices for different numbers of clusters. B: Relative change in the area under the CDF of consensus matrices to choose optimal number of clusters. Red dashed line indicates a 10% change.
